# Transforming LGBTQ+ Aging: From Loneliness to Resilience

**DOI:** 10.1093/geroni/igaf122.001

**Published:** 2025-12-31

**Authors:** Christine Happel, Haley Sanner, Erin Burk-Leaver

## Abstract

Lesbian, gay, bisexual, transgender, queer/questioning (LGBTQ+) older adults in the United States face unique and compounded health and social challenges, including loneliness and social isolation, that compromise the quality of their aging experience. This symposium employs quantitative, qualitative, and mixed methods to provide a comprehensive understanding of the challenges and resilience strategies among LGBTQ+ older adults. The first presentation, utilizing data from the 2023 Behavioral Risk Factor Surveillance System, explores the heightened risks of loneliness and adverse social determinants of health faced by LGBTQ+ older adults. The study highlights the disparities in loneliness, food insecurity, housing insecurity, and access to medical care between LGBTQ+ and non-LGBTQ+ older adults. The second session explores the unique difficulties faced by older lesbians, focusing on their experiences of loneliness and isolation. This study investigates protective factors like community and social engagement to outline possible policy and programming adaptations to support aging well. The third presentation showcases the impact of the ‘Aging Positively’ program for older people living with HIV, particularly long-term survivors. This evaluation explores resilience, self-compassion, survivorship, stigma, and intersectionality. The final presentation assesses the inclusion of LGBTQ+ older adults in state plans on aging. This study uses critical discourse analysis to review documents from all 50 states and the District of Columbia, aiming to create a publicly accessible report card on LGBTQ+ inclusion. Bridging together national data, qualitative narrative, program implementation and policy. These studies offer valuable insights and practical approaches to support the well-being of this diverse and resilient population.

